# A psychometric study of the Russian-language version of the “Bayley Scales of Infant and Toddler Development–third edition”: An assessment of reliability and validity

**DOI:** 10.3389/fpsyg.2022.961567

**Published:** 2022-09-02

**Authors:** Polina Pavlova, Dmitry Maksimov, Dmitry Chegodaev, Sergey Kiselev

**Affiliations:** ^1^Laboratory for Brain and Neurocognitive Development, Department of Psychology, Ural Institute of Humanities, Ural Federal University, Yekaterinburg, Russia; ^2^Department of Preventive and Family Medicine, Ural State Medical University, Yekaterinburg, Russia

**Keywords:** Bayley-III, Bayley Scales of Infant and Toddler Development, psychometric study, child development, confirmatory factor analysis

## Abstract

**Introduction:**

The Bayley Scales of Infant and Toddler Development–third edition (Bayley-III) is one of the most widely used tools for assessing child development, and adapted versions of this instrument have been successfully used in many countries. No comprehensive psychometric studies of the Bayley-III have yet been performed in Russia.

**Materials and methods:**

This psychometric study was part of the longitudinal study conducted by the Ural Federal University in 2016–2020. Within the project, the original Bayley-III manual was translated into Russian and then used in a cohort of 333 infants to assess cognition, expressive/receptive communication, and fine/gross motor skills. For the purpose of psychometric analysis, we selected the data for four age groups of children from the longitudinal study database: 4–6 months (*N* = 149), 10 months (*N* = 138), 15 months (*N* = 151), and 24 months (*N* = 124). The development scores of the sample children were compared with the original Bayley-III norms in each age strata separately. Reliability and validity of the translated instrument were examined using correlation analysis, tests of internal consistency, and confirmatory factor analysis (CFA).

**Results:**

The average scaled scores of the examined children were generally comparable with the original (US) Bayley-III norms, with the exception of those older than 1 year, who demonstrated 1.2–1.9 points better performance in cognitive development and gross motor skills and 0.9–2.6 points lower performance in expressive communication. The correlation of both raw and scaled scores between different scales was low to moderate in all age groups (Spearman’s ρ mostly within the range of 0.3–0.6; *p* < 0.001 for all pairwise correlations). Internal consistency tests confirmed high reliability of the translated instrument (Cronbach’s α = 0.74–0.87, McDonald’s ω = 0.79–0.89). CFA demonstrated a good fit of the three-factor model (cognitive, communicative, and motor components) in all age strata.

**Conclusion:**

The Russian version of the Bayley-III proved to be a psychometrically valid and reliable tool for assessing child development, at least in a research context. The development of the examined children was close to the original US norms, with some deviation in cognitive, gross motor, and expressive communication scores mostly in older children, which could be attributed to the biased sample.

## Introduction

The Bayley Scales of Infant and Toddler Development is one of the most widely used tools for assessing child development. It uses an efficient administration design that relies on age-based starting points, reverse principle, and discontinue criteria. There are four editions of this instrument to date. The latest edition (IV) is considered to have the best clinical sensitivity and accuracy (Bayley and Aylward, 2019), but it has not been thoroughly studied in populations other than US children. Therefore, third edition (Bayley-III) is still widely used around the world as the “gold standard” for assessing the development of children from birth to 3.5 years of age (Bayley, 2006; Yue et al., 2019; Del Rosario et al., 2021). The instrument includes an assessment of five development domains: cognitive, communicative (receptive and expressive communication), motor (fine and gross motor skills), socio-emotional, and adaptive behavior. Although the Bayley scales were originally created for the assessment of US children, the instrument has proven to be a reliable and valid tool in other countries after the linguistic, social, and cultural adaptation (Hanlon et al., 2016; Azari et al., 2017; Ranjitkar et al., 2018; Hua et al., 2019; McHenry et al., 2021). The domains of adaptive behavior and socio-emotional development were rarely used in psychometric research due to the complexity of cross-cultural adaptation (McHenry et al., 2021), so the majority of the studies focused primarily on the cognitive, communication, and motor skills assessment.

The majority of Bayley-III studies were conducted under umbrella of the larger child development research (Hanlon et al., 2016; Azari et al., 2017; Ranjitkar et al., 2018). Most often, they included healthy children, but also, depending on scientific goals, children with clinical diagnoses could participate. In most cases, after appropriate translation considering local cultural, historical, and social features, the instrument showed to be valid and reliable in different cultures (Sun et al., 2019; McLester-Davis et al., 2021; Salah El-Din et al., 2021). Some non-US studies demonstrated successful application of the Bayley-III using the original norms even without prior socio-cultural adaptation (Yu et al., 2013; Ballot et al., 2017).

After translation and cultural adaptation of the psycho-diagnostic tool, it is necessary to assess whether the implied latent constructs were preserved. Various approaches can be used to evaluate the psychometric quality of the adapted tool: indirect comparison with the original (US) norms, assessment of internal consistency, test-retest and inter-rater reliability, and study of construct and criterion validity (Azari et al., 2017; Hua et al., 2019; Sun et al., 2019; McHenry et al., 2021). A reference method for assessing construct validity is confirmatory factor analysis (CFA). It is a specially designed statistical approach to study a correlation between some implied latent psycho-physical factor(s), predefined in a specific set of questions or tests, and an actual data. Although considered the most accurate, CFA is still underused in the literature due to its overall complexity and the need for special software (Madaschi et al., 2016; Sun et al., 2019; McHenry et al., 2021).

Within CFA, when assessing cognitive, communicative and motor development, the Bayley-III implies three-factor model when each of these domains or latent factors corresponds to a separate scale: cognitive factor (cognitive scale), a communicative factor (subtests of receptive and expressive communication), and a motor factor (subtests of fine and gross motor skills). This model is generally used as a reference, although less differentiated factor combinations are also considered appropriate, especially in non-original cultural settings The one-factor model included the results of all five scales as indicators of a common developmental factor (Azari et al., 2017; Sun et al., 2019). The two-factor model included a cognitive-communicative factor (cognitive scale, subtests of receptive, and expressive communication) and a motor factor (subtests of fine and gross motor skills) (McHenry et al., 2021). In some studies, several factor structures were analyzed simultaneously within a single dataset (Madaschi et al., 2016; McHenry et al., 2021).

**FIGURE 1:**
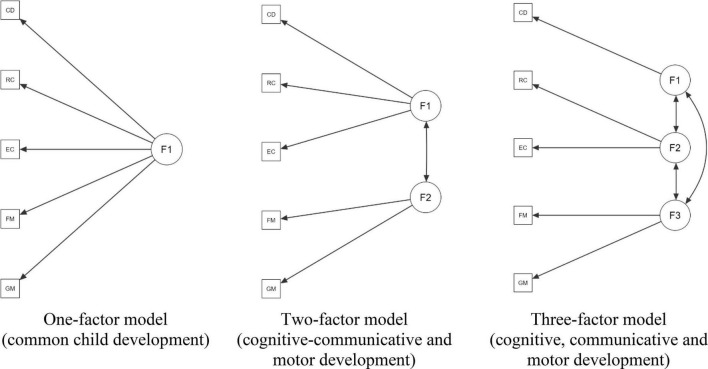
Theoretical models of Bayley-III factor structure. CD, cognitive development; RC, receptive communication; EC, expressive communication; FM, fine motor; GM, gross motor.

Three theoretical models of the Bayley-III factor structure are shown in Figure 1.

There is no single standard for assessing child development in the Russian Federation. In practice, several diagnostic scales are used, and the final choice of method depends on a specialist’s personal preference, which makes it difficult to compare the results of different observations or monitor child development over time in a coherent manner (Kosenkova et al., 2012). The most used methods for quantifying child development are translated version of The Griffith Mental Development Scales, 2nd edition of the Bayley Scales of Infant Development (Bayley, 1993), and the original Russian tool (so called «GNOM») specially designed to assess neuropsychological development of infants (Kozlovskaja et al., 2017).

All above listed methods have some limitations and none of them was thoroughly tested in Russia for psychometric quality in accordance with modern scientific requirements. Thus, there is a call for the universal, up-to-date child development diagnostic tool and the Bayley-III is one of the best candidates to fill this gap. It is based on the well-known and widely accepted developmental theories (D. Bruner, L. S. Vygotsky, A. R. Luria, J. Piage) and is consistent with the findings of large studies of child development (Greenspan et al., 2001; Colombo and Mitchell, 2009; Weiss et al., 2010). Bayley-III assesses the main areas of child development with high accuracy during the entire period of younger childhood starting from the first days of life. The psychometric properties of Bayley-III were validated according to the best practices in the field (AERA et al., 2014).

To date the instrument had not been fully adapted in Russia and has been used only in small experimental studies (Bakushkina et al., 2018; Belousova and Shvets, 2019). In most cases, the authors used US norms as the reference standards for assessing Russian children (Shifman, 2016; Zavadenko et al., 2018; Kosyakova and Bespalova, 2019). To overcome these limitations and facilitate further adaptation of the Bayley scales, at the first stage we performed an in-depth analysis of the scale structure, the original standardization procedure, and international experience of adaptation/psychometric validation of the instrument. Then we conducted a pilot study of the Bayley III on 163 healthy Russian infants aged 2–11 months, which demonstrated a high similarity of all neurodevelopment indicators to the original US norms (Pavlova et al., 2020). At the next stage, described in the current article, we conducted a comprehensive psychometric study of the Bayley-III cognitive, communication and motor scales in different age samples of Russian children from the city of Yekaterinburg.

## Materials and methods

### Participants

The study was a part of the project “Longitudinal study of the neurocognitive development of children with perinatal trauma,” which was conducted by the Department of Psychology Laboratory of Brain and Neurocognitive Development (Ural Federal University, UrFU, Yekaterinburg) from 2016 to 2020. The study protocol was approved by the ethics committee of the Ural State Medical University (Yekaterinburg). Written informed consent to participate in the study was provided by the children’s parents or next of kin. The longitudinal cohort included mostly healthy children born at physiological gestational age, as well as premature infants, children with a history of neurological disorders of varying severity (perinatal CNS damage, ischemic stroke, psycho-motor retardation), and children at risk of autism spectrum disorder (ASD) or attention deficit hyperactivity disorder (ADHD). The majority of parents of the examined children represented the educated urban population of Yekaterinburg and the Sverdlovsk region. The longitudinal study database, which included development indicators obtained from the observation of 333 children, was used as a source of the data for this psychometric study.

Only children with a complete Bayley-III assessment were included in the psychometric analysis. Individual records were selected from the longitudinal study database in accordance with the age ranges of the original Bayley-III with at least 100 children in each age group. The youngest age stratum combined the age points of 4, 5, and 6 months. If a child was assessed more than once in the same age period, only one observation with the complete medical, demographic, and psychometric data was included in the analysis. Thus, from the total data pool of children observed in the longitudinal study, four age groups were created: 4–6 months (3 months 16 days–6 months 22 days; *N* = 149), 10 months (9 months 0 days–10 months 30 days; *N* = 138), 15 months (13 months 16 days–16 months 15 days; *N* = 151), and 24 months (22 months 16 days–25 months 15 days; *N* = 124). A total of 562 unique assessments were used. The detailed clinical and demographic characteristics of the children in each age group are presented in Table 1.

**TABLE 1:** Clinical and demographic characteristics of the examined children.

Characteristic	4–6 months (*N* = 149)	10 months (*N* = 138)	15 months (*N* = 151)	24 months (*N* = 124)	Cramer’s V	*p*
Boys	56%	59%	57%	54%	0.03	0.9
Healthy children	44%	46%	45%	40%	0.04	0.8
Family history of ASD	8%	10%	12%	9%	0.04	0.8
Family history of ADHD	2%	2%	3%	3%	0.03	0.9
Premature infants	29%	25%	17%	14%	0.14	0.01
History of ischemic stroke	12%	15%	21%	28%	0.15	0.004
Other neurological pathology	5%	2%	2%	5%	0.08	0.3

The clinical and demographic characteristics of the children were pretty similar in all age groups, except for the older ones, where there were fewer children with a history of prematurity but more with a history of ischemic stroke.

### Instrument and procedures

The Bayley-III instrument includes five scales with a definite number of diagnostic items\tasks: (a) cognitive scale (91 items); (b) language scale with subscales of receptive language (49 items) and expressive language (48 items); (c) motor scale with subscales of fine motor (66 items) and gross motor (72 items); (d) social-emotional scale (35 items); and (e) adaptive behavior scale (241 items). Cognitive, language, and motor scales directly assess a child’s performance on items. If a child performs the items correctly, one point is scored. Then, all item points are summed up for each scale.

Translation of the Bayley-III evaluation forms and stimulus materials was performed with the preservation of the original contextual meaning. Difficulties in the modification of the tool were primarily associated with the grammatical differences in English and Russian languages. For example, in the items 34 from the subscales of receptive and expressive communication, present continuous tense was translated as present simple, which sounded more natural in the Russian language in this particular context. In item 34, the subscale of receptive language, and item 34, the subscale of expressive language, the original version assumes an assessment of a child’s understanding and use of the present continuous, which has no direct analogue in Russian speech. Instead, when describing static situations and events occurring now, the present simple tense is preferable and was actually used during diagnostics. In this regard, after the translation these items were modified in the following manner: item 34 in the subscale of receptive language: (eng) Understands Verb + ing → (rus) «Понимaeт глaгoлы B нacтoящeм вpeмeни» item 34 in the subscale of expressive language: (eng) Uses Verb + ing → (rus) «Иcпoльзyeт cyщecтвитeльнoe, oпиcывaющee дeйcтвиe».

Translated version was used to assess cognitive, communicative and motor development of the children in the first 2 years of life. The laboratory researchers, who made the assessments, had been received comprehensive training by certified Bayley-III specialist from the United Kingdom. Diagnostic environment was organized according to the Bayley-III standards in a specially equipped room. Children were tested in the presence of their parents/caregivers, who were instructed about the procedure. When needed, assessments were interrupted for feeding, rest, or napping. Children with motor impairments used their most functional limb during the examination. All procedures were video recorded for further analysis.

### Statistical analysis

Bayley-III psychometric indicators were presented as raw and scaled scores. Only scores with data on all 5 scales were used for the analysis. Statistical procedures were performed in Jamovi (R) 2.2.5 (AGPL-3.0 license) and Gretl 2021b (GPL license) statistical software. Student’s *t*-test was used for indirect comparison of the mean scaled scores with the original US standard values (10 ± 3 points).

#### Reliability assessment

The Spearman’s rank correlation coefficient was used to study pairwise correlations between Bayley-III raw and scaled scores in all age strata separately. The following guidelines were adopted for interpreting the magnitude of the correlation: very high (0.90–1.00), high (0.70 –0.90), moderate (0.50–0.70), low (0.30–0.50), and negligible (0.00–0.30) (Mukaka, 2012). Cronbach’s α and McDonald’s ω were used to calculate internal consistency, and a value > 0.7 was considered a sign of high reliability.

#### Validity assessment

The CFA method (Suhr, 2006) was used to test the putative Bayley-III factor structure. Three theoretical models of the Bayley-III factor structure were evaluated and compared in all age groups. The one-factor model included the results of all five scales as indicators of a common developmental factor. The two-factor model included a cognitive-communicative factor (cognitive scale, subtests of receptive, and expressive communication) and a motor factor (subtests of fine and gross motor skills). The three-factor model consisted of a separate cognitive factor (cognitive scale), a communicative factor (subtests of receptive and expressive communication), and a motor factor (subtests of fine and gross motor skills). The following statistical criteria were used to evaluate the accuracy of the CFA models: (1) ratio χ2/df; where the indicator <2 indicated the model’s high accuracy, <5–acceptable accuracy; (2) *p* > 0.05 in the χ2 test indicates a good fit; (3) Root Mean Square Error of Approximation (RMSEA) < 0.05 indicates good fit, 0.05–0.08–acceptable fit; (4) Comparative Fit Index (CFI) ≥ 0.95 indicates a good fit; ≥ 0.90—acceptable fit; (5) Standardized Root Mean Square Residual (SRMR) with good fit < 0.08; (6) Tucker Lewis Index (TLI) ≥ 0.95 indicates a good fit; and (7) Akaike information criterion (AIC) and the Bayesian information criterion (BIC) for which smaller is better, were used to compare models with each other. The correlation between individual Bayley-III scales and the corresponding latent factors was assessed using standardized factor loadings; a coefficient of 0.5 was considered acceptable, while a coefficient of 0.7 and above was considered good.

To achieve 80% power in CFA with acceptable accuracy (RMSEA ≤ 0.08) when studying three-factor model of the Bayley-III, one should sample approximately 600 participants (Moshagen and Erdfelder, 2016). Due to the natural restrictions, we were not able to recruit such number of children; nevertheless, we kept this analysis in the protocol due to its overall importance.

## Results

The results of the children’s development assessment on each Bayley-III domain are presented in the scaled scores. Mean scores were compared to the original (US) norms using a *t*-test (Table 2).

**TABLE 2:** Scaled scores of child development.

Subtest	4–6 months			10 months			15 months			24 months		
	Mean	SD	*p*	Mean	SD	*p*	Mean	SD	*p*	Mean	SD	*p*
Cognitive	10.7	2.4	0.008	10.7	2.3	0.05	11.6	2.7	<0.001	11.2	3.9	0.01
Receptive c.	9.1	1.9	<0.001	9.7	2.5	0.4	10.5	2.7	0.2	10.3	3	0.5
Expressive c.	10.2	2	0.4	10	2.2	1	9.1	1.6	0.007	7.4	2.3	0.001
Fine motor	9.3	2.3	0.007	9.7	3	0.5	10.9	3.1	0.02	10.3	3.2	0.5
Gross motor	9.8	2.5	0.5	9.7	1.9	0.4	11.9	2.9	<0.001	11.4	3.1	0.001

The average scaled scores were close to the original (US) Bayley-III standard scores with some exceptions. Cognitive development in all age groups and gross motor skills in older children were moderately higher, whereas the development of expressive communication in older children was significantly lower.

The pairwise correlations between different development indicators were calculated in each age group using Spearman’s rank coefficient (due to the non-normality of the data). Both for the raw and scaled scores in most cases the correlation ranged from low to moderate (0.3–0.6). All correlations were statistically significant with *p* < 0.001. An example of the correlation matrix in 24 months age group is presented in Table 3.

**TABLE 3:** Correlation matrix of the scaled scores in 24 months age group.

Subtest	Cognitive	Receptive communication	Expressive communication	Fine motor	Gross motor
Cognitive	–				
Receptive communication	0.623	–			
Expressive communication	0.527	0.564	–		
Fine motor	0.497	0.539	0.524	–	
Gross motor	0.603	0.534	0.425	0.492	–

### Reliability

The ranges of internal consistency coefficients for the five scales in all age groups were in the range of 0.641–0.866 for Cronbach’s alpha and 0.707–0.883 for McDonald’s omega (Table 4). For both coefficients value > 0.7 was considered a sign of high reliability.

**TABLE 4:** Internal consistency assessment.

Subtest	4–6 months		10 months		15 months		24 months	
	α	ω	α	ω	α	ω	α	ω
Cognitive	0.775	0.829	0.641	0.707	0.720	0.748	0.834	0.841
Receptive communication	0.857	0.879	0.703	0.752	0.762	0.794	0.829	0.854
Expressive communication	0.866	0.905	0.696	0.750	0.797	0.819	0.862	0.883
Fine motor	0.754	0.829	0.699	0.759	0.707	0.729	0.845	0.856
Gross motor	0.787	0.854	0.749	0.772	0.752	0.776	0.847	0.866

### Validity

Confirmatory factor analysis with raw subtest scores was used to study the construct validity of the one-, two- and three-factor models of child development in all age groups. The goodness-of-fit criteria for each of the three models are presented in Table 5.

**TABLE 5:** Goodness-of-fit indices from CFA models by age groups.

Model	χ2df	χ2/df	*p*	CFI	TLI	SRMR	RMSEA	AIC	BIC
**4–6 months**									
1 Factor	5.75	1.14	0.34	0.998	0.997	0.0166	0.031	3176	3221
2 Factors	5.74	1.43	0.22	0.996	0.991	0.0168	0.053	3178	3226
3 Factors	5.63	1.86	0.13	0.994	0.981	0.0165	0.076	3180	3231
**10 months**									
1 Factor	22.05	4.4	<0.001	0.909	0.818	0.0534	0.157	3181	3225
2 Factors	21.34	5.33	<0.001	0.907	0.768	0.0519	0.177	3182	3229
3 Factors	4.93	1.63	0.178	0.990	0.966	0.0321	0.0680	3168	3218
**15 months**									
1 Factor	8.705	1.74	0.122	0.985	0.969	0.0337	0.0700	3776	3821
2 Factors	8.514	2.13	0.075	0.981	0.953	0.0333	0.0864	3778	3826
3 Factors	4.223	1.41	0.239	0.995	0.983	0.0203	0.0519	3775	3827
**24 months**									
1 Factor	13.35	2.66	0.021	0.975	0.949	0.0297	0.116	3507	3549
2 Factors	6.394	1.59	0.172	0.993	0.982	0.0199	0.0694	3502	3547
3 Factors	4.133	1.38	0.247	0.997	0.988	0.0173	0.0552	3502	3550

The three-factor structure of the Bayley-III was confirmed in all age groups and had a good fit according to the CFI and SRMR indicators. RMSEA indicators in all cases had acceptable values. All three types of models had a good fit among children of 4–6 months. The one-factor model also had a good fit at 15 months and the two-factor model at 24 months; however, they were less consistent than the reference three-factor model.

The correlation between the Bayley-III subtests and latent factors, which are expressed as standardized factor loadings (SFL), and the proportion of the indicator variability explained by the latent factor (*R*^2^), were calculated in all age groups for the three-factor model (Table 6).

**TABLE 6:** Standardized factor loadings for a three-factor model.

Subtest	4–6 months		10 months		15 months		24 months	
	SFL	*R* ^2^	SFL	*R* ^2^	SFL	*R* ^2^	SFL	*R* ^2^
Cognitive	1	1	1	1	1	1	1	1
Receptive communication	0.66	0.43	0.74	0.55	0.68	0.46	0.86	0.74
Expressive communication	0.50	0.25	0.75	0.56	0.54	0.29	0.68	0.46
Fine motor	0.93	0.87	0.72	0.52	0.86	0.74	0.87	0.76
Gross motor	0.81	0.66	0.53	0.28	0.65	0.42	0.77	0.59

Most of the SFLs were equal to or greater than 0.5, which indicated an acceptable and good correlation between the Bailey-III subtests and the latent factors of child development.

## Discussion

In this study, we evaluated the psychometric quality of the translated version of the Bayley-III on a sample of Russian children representing relatively well-educated families from urban areas. To our knowledge, this is the first study that examined the reliability and validity of the Bayley-III in the Russian Federation. The results of using Bayley-III for Russian children complement the international practice of using the tool in different sociocultural conditions (Krogh et al., 2012; Ballot et al., 2017; Salah El-Din et al., 2021). Despite a relatively high proportion of children with a history of prematurity and transient neurological disorders in the study sample, development indicators were similar to the original US norms for healthy children, with even better performance on the cognitive scale in all age groups. These results could be in part explained by the biased sample from urbanized areas and relatively well-educated families. It was also possible that many parents were seriously concerned about neurodevelopment of their children, which motivated them to join the study. This could potentially influence the representativeness of the sample as well. As reported previously, parental concern is the significant factor influencing child development and these factors need to be taken into account in research in developmental psychology (Algarvio and Leal, 2016). Additionally, older children tend to have better gross motor skills and lower scores on expressive communication. It is also should be interpreted more because of the biased sample rather than reflection of the population characteristic. For example, a study of speech development of Russian children, which used the validated MacArthur-Bates Communicative Development Inventories, demonstrated higher scores compared to the US norms (Eliseeva et al., 2016). Finally, revealed normal indicators in the relatively morbid sample could reflect the known Bayley-III tendency to overestimate development, resulting in an under-identification of children with developmental delay (Anderson and Burnett, 2017). Nonetheless, development of Russian children were very close to the US norms, which may indicate substantial physiological and cultural similarity.

The reliability of Russian Bayley-III was studied using Cronbach’s alpha. The majority of previous studies demonstrated high reliability (≥0.7) of this coefficient in all Bayley’s scales (Zakaria et al., 2012; Madaschi et al., 2016; Azari et al., 2017). According to the growing body of the literature, the reliability indicators may be influenced by the sample size and the age of the children. Usually, higher reliability rates were obtained in studies where sample of children included all age groups (Madaschi et al., 2016; Azari et al., 2017). In our study analysis of internal consistency was performed in all age strata separately. A similar approach was used in the original Bayley-III study and in the study of the Vietnamese children (Bayley, 2006; Sun et al., 2019). Most scales across all ages have been shown to be reliable (≥0.7). In children of older age groups, the indicators of internal consistency were slightly higher (≥0.8), which is consistent with the results of international studies (McHenry et al., 2021; McLester-Davis et al., 2021). Relatively low reliability coefficients were recorded on the communicative scale at 10 months (<0.7). This could be because the communicative function is the most specific reflection of a particular culture; thus, when translated into another language, a psychometric tool may not fully correspond to the original. Similar results were also observed in the studies of Vietnamese and Nepalese children (Ranjitkar et al., 2018; Sun et al., 2019), that could be explained by some similarity in communicative culture of Russia and these countries. In accordance with Hall’s context theory (Hall and Hall, 1989), there are two different categories of cultures—high context and low context. The high-context communication is characterized by less information is in the verbal message and more in the context, non-verbal communication (eye contact, facial expressions, gestures, tone of voice, etc.). In contrast, the low-context communication assumes that most of the information is in the verbal message (spoken words, written notes, etc.) and less in the context (Nam, 2015). According to this theory, the US is considered a low context culture, while Vietnam, Nepal, and Russia belong to the high context.

Reliability analysis using McDonald’s omega was also used in our study. This indicator is considered more accurate since it contains the assumption of the model’s tau equivalence (Dunn et al., 2014). This coefficient has not been used in the Bayley-III studies but has been shown to be useful in studies of other diagnostic methods in medicine and psychology (Hayes and Coutts, 2020; Weyn et al., 2021; Fung et al., 2022). In this study, high reliability in terms of the omega coefficient was revealed on all scales in all age groups, including indicators of speech development. Overall, the high reliability scores of the Russian-language Bayley-III, which were obtained from internal consistency analyses using multiple approaches, indicated that all tests for each of the five scales were capable of reliably detecting all putative latent factors (developmental indicators). In other words, the instrument completely retained its psychometric properties after being transferred to a Russian-speaking environment. Most of the psychometric studies focused on Bayley-III also demonstrated good and acceptable internal consistency, which indicates the high quality of the tool and its potential for transfer to a new cultural environment.

The assessment of construct validity using confirmatory factor analysis was an important component of our psychometric study. The initially postulated Bayley-III factor structure was reliably identified by the Russian-language version based on a given set of psychometric indicators, even though the study was somewhat under-powered for this type of analysis. The neuropsychic development of children manifested itself mainly in the form of three main domains (cognitive, communicative, and motor), which fully corresponded to the original psychometric structure of Bayley-III (Bayley, 2006). We also found a good fit of the one- and two-factor models in several age strata. Such findings could be explained from the viewpoint of child development theory (Sun et al., 2019). For example, a study by McHenry et al., based on several Bayley-III psychometric studies in different populations, found that the number of leading factors may vary depending on the age of the children (McHenry et al., 2021). Good consistency of one and two factor models at younger ages may indicate a closer relationship between cognitive, speech, and motor development during infancy, when these manifestations of higher nervous activity are at the early stage of differentiation, and the development itself is largely determined by the physiologically universal processes. As a child grows up, his psycho-physical manifestations become more complex, social, and cultural influences make the development trajectory more individualized, which is naturally revealed in psychometric analysis as the dominance of a more differentiated three-factor model.

However, there were studies where one-factor model showed good consistency in older children and, on the contrary, was not confirmed in younger age groups (Sun et al., 2019). In some studies, where the samples included children of different ages, the one-factor model also demonstrated the best fit (Madaschi et al., 2016; Azari et al., 2017). Thus, despite the common patterns of child development and the similarity of psychophysical manifestations in different populations, under the influence of complex social, cultural, and ethnic characteristics, diagnostic tool does not always perform as in the original population. In our study, the identified three-factor model was the most psychometrically appropriate for the Bayley-III method in the Russian language, when, in accordance with the results of the factor loading assessment, the communicative scale is loaded with one factor, expressive and receptive communication are loaded with another factor, and fine and gross motor skills are loaded with a third factor.

## Strengths and limitations of the study

Our study was the first that examined psychometric quality of the Russian version of the Bayley-III according to the current research standards in each step, from translation to statistical analysis. For validity assessment we used confirmatory factor analysis–an advanced statistical technique, that showed promising results even on the relatively small sample. We also confirmed the predefined three-factor structure of the Bayley-III in different age groups and revealed some similarities in development patterns of Russian children and their peers from other countries.

The main limitation of this study was the biased sample (educated urban population, relatively high proportion of children with a history of prematurity and neurological disorders), so one should generalize the results with caution. The number of observations in each age group (from 124 to 151) was sufficient to analyze reliability and perform indirect comparison with the original indicators but was relatively small for validity assessment using CFA. We also used third edition of the Bayley scales, while the fourth edition has already been available since 2019 and is considered to have better psychometric quality and clinical sensitivity. Thus, our results might not demonstrate the best accuracy and could be affected by the known tendency of Bayley-III to underestimate developmental delay.

## Conclusion

After linguistic adaptation to a Russian-speaking environment, the Bayley-III scales proved to be a reliable and valid tool for studying child development in the age range from 4 to 24 months. Confirmation of construct validity and good internal consistency both indicated high quality of the Russian translation and the psychometric quality of the instrument *per se*. These results supported the wider use of the Russian Bayley-III, at least in a research context, and considered the potential of this instrument as a reliable clinical diagnostic tool. The results of this study could also be applied to the Russian-speaking children (families) outside Russia, in countries with similar cultural context since we did not change the original stimulus material.

The developmental indicators of the examined children in general were close to the original Bayley-III norms, with slightly better performance on the cognitive scale in all age groups but some delay of expressive communication in older children. These differences should be attributed more to the biased sample rather represent true linguistic and cultural features of Russian children. In any case, further multi-center studies of a larger sample sizes are recommended to clarify the results.

## Data availability statement

The raw data supporting the conclusions of this article will be made available by the authors, without undue reservation.

## Ethics statement

The studies involving human participants were reviewed and approved by Local Ethics Committee of the Ural State Medical University of the Ministry of Health and Social Development of the Russian Federation. Written informed consent to participate in this study was provided by the participants or their legal guardian/next of kin.

## Author contributions

DM and PP contributed to the conception and design of the study and performed the statistical analysis. DC conducted the analysis of medical data as a neurologist. SK organized the database. All authors wrote sections of the manuscript, contributed to the manuscript revision and read, and approved the submitted version.
